# ‘Lint ball’ omphalitis, a rare cause of umbilical discharge in an adult woman: a case report

**DOI:** 10.4076/1757-1626-2-7785

**Published:** 2009-07-27

**Authors:** Deba P Sarma, Bryan Teruya

**Affiliations:** Department of Pathology, Creighton University Medical CenterOmaha, NE 68131USA

## Abstract

**Introduction:**

Umbilical discharge in adult is rare and is usually induced by foreign material, most commonly hair. Rarely, it may be due to embryonal anomalies. We are reporting an unusual case of umbilical discharge in adult secondary to an impacted lint ball.

**Case presentation:**

A 55-year-old obese woman presented with a 4-month history of hemorrhagic discharge from the umbilicus. Deep from the base of the umbilicus, a 0.8 cm gray-tan mass was removed that on microscopic examination revealed a lint ball.

**Conclusion:**

An impacted lint ball may be a rare cause of umbilical discharge in adult.

## Case presentation

**Figure 1.**
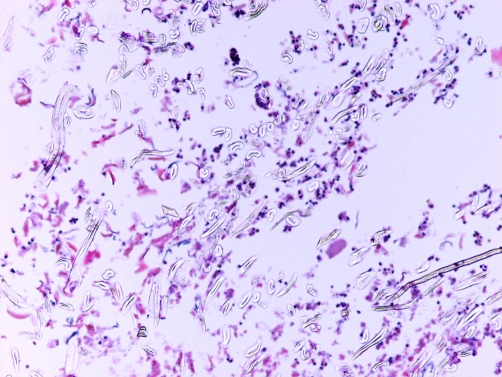
Low power photomicrograph shows refractile lint material, keratin and neutrophils.

**Figure 2.**
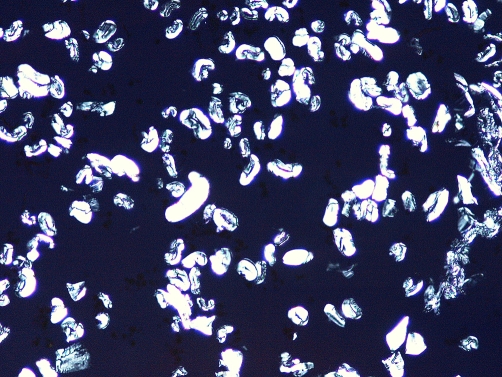
Lint ball under polarized light.

A 55-year-old obese white American woman of European descent presented with a 4-month history of slightly hemorrhagic discharge from her umbilicus. There was no history of fever, abdominal pain or any other systemic disease. Physical examination revealed a deep umbilicus with a barely visible opening. There was no redness, edema, or crusting of the periumbilical skin. The deeper aspect of the umbilicus was exposed by using a spatula. A dark, rounded polypoid mass was noted. The clinical impression was that of fibro-epithelial polyp or some other tumor. An attempt was made to remove the mass by excising the base; however, the mass easily came out of the umbilical cavity implying that either it was necrotic or it was not firmly attached to the umbilical tissue at the base. The gray-tan 0.8 cm size round mass on cut section revealed white fibrous appearance. On microscopic examination, it was composed of lint material with typical morphology of refractile bean-shaped and elongated colorless structures, red spindle-shaped keratin material, granular red debris, rare hair fragments and polymorphonuclear leukocytes (Figure 1). Under polarized light, the lint particles showed brilliant blue-green birefringence (Figure 2). A diagnosis of ‘lint ball’ omphalitis was made.

The patient remained completely asymptomatic at the follow-up visit one month later.

## Discussion

Umbilical discharge in adult is rare but can be quite alarming. It may be caused by various congenital or acquired conditions. Patients with embryonal anomalies, such as patent urachus, urachal cyst or sinus, patent vitelline duct, vitelline cyst or sinus may present as umbilical discharge [1]. However, the most common cause of umbilical discharge in adult is acquired conditions, such as pilonidal sinus disease [2,3], infection due to hair tufts and foreign bodies [4], and non-specific acute and chronic inflammation and abscess of the umbilicus [5]. Very rare causes include endometriosis and metastatic carcinoma [4].

The present case is definitely a foreign body-induced omphalitis. Hairball is the most common type of foreign body seen in such cases. Most of the patients are young, hairy male with deep umbilicus with poor personal hygiene [2]. One interesting report of foreign body-induced umbilical discharge, similar to the present case was that of a 47-year-old obese female with an old toilet paper ball in the umbilicus [1].

We could not find any reported case of ‘lint ball omphalitis’.

Finding of ‘belly-button lint’ is quite common among hairy man. Usually it is washed off during bathing or shower and rarely does it cause any inflammation. Steinhauser has recently suggested that abdominal hair is mainly responsible for directing the fibers from clothes into the navel where they are compacted [6]. Shaving abdominal hair can prevent lint accumulation in the umbilicus.

Obesity, deep umbilicus, and poor hygiene may have been the predisposing factors for developing lint accumulation and subsequent omphalitis in our patient.
